# Effect of Maternal Gradient Nutritional Restriction during Pregnancy on Mammary Gland Development in Offspring

**DOI:** 10.3390/ani13050946

**Published:** 2023-03-06

**Authors:** Xusheng Dong, Xueyan Lin, Qiuling Hou, Zhiyong Hu, Yun Wang, Zhonghua Wang

**Affiliations:** College of Animal Science and Technology, Shandong Agricultural University, Taian 271018, China

**Keywords:** mammary development, nutritional restrictions, pregnancy, offspring

## Abstract

**Simple Summary:**

The embryonic period, together with puberty and pregnancy, are known as the three main stages of mammary gland development. The development of the mammary glands is slowed during the embryonic period due to factors such as inadequate nutrition, which directly affect the development of the mammary glands and lactation after birth. However, the impact of embryonic nutrition on fetal mammary gland development is often unnoticed. We investigate the effect of nutritional intake on embryonic mammary gland development by administering different levels of nutritional restriction to female mice during gestation. Contrary to common belief, we found that mild maternal nutritional restriction contributes to mammary gland development in the offspring. Mammary gland dysplasia is not obvious until maternal nutritional restriction reaches 70% of the normal intake. Further embryonic mammary gland development studies can be performed based on our level of maternal nutritional restriction. In addition, the use of mice as model animals can also provide a reference for dairy farming, where nutrition should not be excessive during the gestation period of the cow; otherwise, it affects the mammary gland development of the offspring.

**Abstract:**

We aimed to investigate the effect of different levels of nutritional restriction on mammary gland development during the embryonic period by gradient nutritional restriction in pregnant female mice. We started the nutritional restriction of 60 female CD-1(ICR) mice from day 9 of gestation based on 100%, 90%, 80%, 70% and 60% of ad libitum intake. After delivery, the weight and body fat of the offspring and the mother were recorded (*n* = 12). Offspring mammary development and gene expression were explored by whole mount and qPCR. Mammary development patterns of in offspring were constructed using Sholl analysis, principal component analysis (PCA) and regression analysis. We found that: (1) Mild maternal nutritional restriction (90–70% of ad libitum intake) did not affect offspring weight, while body fat percentage was more sensitive to nutritional restriction (lower at 80% ad libitum feeding). (2) A precipitous drop in mammary development and altered developmental patterns occurred when nutritional restriction ranged from 80% to 70% of ad libitum intake. (3) Mild maternal nutritional restriction (90% of ad libitum intake) promoted mammary-development-related gene expression. In conclusion, our results suggest that mild maternal nutritional restriction during gestation contributes to increased embryonic mammary gland development. When maternal nutritional restriction reaches 70% of ad libitum intake, the mammary glands of the offspring show noticeable maldevelopment. Our results help provide a theoretical basis for the effect of maternal nutritional restriction during gestation on offspring mammary development and a reference for the amount of maternal nutritional restriction.

## 1. Introduction

Nutritional challenges that occur during gestation, a critical period for embryonic growth and development, may lead to alterations in the physiological development and metabolism of the offspring after birth [1]. The most possible nutritional challenges during gestation are undernutrition and overnutrition, which can affect the health of both the fetus and the maternal body [2]. In particular, malnutrition during pregnancy, which still exists in underdeveloped regions, as a global problem, has important implications for the healthy development of the mother and the newborn [3]. These nutritional damages can cause permanent adjustments in the embryonic physiological state and organ development by inducing genetic changes in the proliferation/differentiation pathways during embryonic development [4,5]. Current research on nutritional restriction during pregnancy has focused on the placenta, brain and other organs that affect fetal survival [6,7], with little attention paid to fetal mammary gland development.

The embryonic period, puberty and pregnancy are known as the three main stages of mammary gland development. The embryonic development of the mammary gland begins in many mammals at mid-gestation [8]; for mice, with a gestation period of 19–21 days, the mammary gland initiates development on day 10 of embryonic life [9]. The embryonic mammary glands are formed by a bilaterally multilayered ectodermal stripe from the forelimb bud to the hindlimb bud on the ventral surface of the embryo, referred to as the mammary line [10]. At day 11.5 of the mouse embryonic stage, these milk lines form five visible pairs of placebos. These placebos then become embedded in the mammary mesenchyme. At day 15–16 of the mouse embryonic stage, primary bud formation invades the secondary mammary mesenchyme and begins to develop a branching morphology [11]. Before birth, the mammary gland consists of a small ductal tree with a dominant duct and 10–15 branches embedded in the nascent fat pad [12]. The basic mammary duct system that forms at this time arises in the absence of hormonal input and remains essentially quiescent until puberty [10]. This basic ductal system forms the framework from which the mammary glands develop further during puberty and pregnancy to form the mature mammary glands [13]. If the development of the embryonic mammary glands is slowed at this period due to nutritional deficiencies and other factors, it directly affects the development of the mammary glands after birth and may even affect the amount of milk produced during lactation [14,15].

Although the embryonic stage is the initiation of mammary gland development, nutritional regulation has remained less studied for this stage of mammary gland development. Since puberty is considered a critical window for nutritional regulation, most nutrition-related research has focused on this period [16,17]. The impact of embryonic nutrition on fetal mammary gland development is often unnoticed. Although the mammary gland that develops during the embryonic period is considered a basic ductal system, it has the ability to produce milk, known as neonatal milk or switch’s milk [10]. This indicates that the mammary gland is already equipped with basic lactation functions after birth, and if there are problems with the development of the mammary gland during the embryonic period, these basic functions are affected. Terminal end buds, an important structure in the extension of the mammary ducts during puberty, form only at the tips of the elongated ducts which are based on branches generated during the embryonic period [9,10]. Multipotent mammary stem cells (MASCs) from embryonic mammary gland formation are the source of MASCs and progenitor cells required for mammary duct development during puberty and alveolar luminal formation during pregnancy [9]. These results all suggest that there is a connection between embryonic, pubertal and gestational mammary development, and that mammary gland damage caused by nutritional fluctuations received during the embryonic period further affect mammary gland development after birth.

The embryonic stage is the period of initial mammary gland formation, when the mammary gland gradually begins to expand through proliferation and differentiation in multipotent MASCs [18]. Many genes associated with mammary stem cells during the embryonic period have been shown to influence the future developmental fate of the mammary gland. The Axin2 gene was found to have the ability to allow cell regeneration in mammary gland transplantation assays, and the expression of the Axin2 gene during the embryonic period has been shown to be associated with future development in the ductal cell lineage [18]. During this period, Wnt5a has also been shown to be required for normal development of the mammary ducts [19]. In addition, MASCs marker genes such as Sox10, Procr, ELF5 and Aldh1a1 were identified by knockout studies and regulate key functions of mammary gland development [20,21,22,23]. After birth, MASCs become lineage-restricted with some becoming progenitor cells and contributing to the development of the mammary gland base or lumen [9]. Thus, nutritionally induced changes in embryonic mammary development may continue to affect mammary development in adulthood. The effect of altered nutrient levels on mammary stem cells has been demonstrated in previous studies with cells and adult mice [24,25]. Maria Theresa E. Montales et al. found that angiotensin in food affects the number of mammary cell-like/progenitor cells [24]. Omar M. Rahal et al. speculated that diet-regulated hormonal signaling could influence MASC self-renewal [25]. Studies on the effects of nutritional restriction on mammary stem cells have had mixed results, with one study suggesting that nutritional restriction attenuates mammary stem cell viability and inhibits mammary gland development [26], while another study suggests that nutritional restriction induces the self-renewal of mammary stem cells [27]. These results may be due to differences in the amount of nutrient limitation. Mild nutrient limitation mediates the restoration of stem cell self-renewal capacity through nutrient and energy-sensing pathways [27]. When nutrient limitation exceeds the regulatory level of the cells, apoptosis and necrosis of stem cells can occur due to nutrient deficiency.

Compared to nutritional treatment after birth, nutritional treatment for the embryonic period is more difficult and requires nutritional interventions for the maternal body. The most common approach in studies of fetal undernutrition is accomplished through food or caloric restriction of the mother during gestation [28]. The mammalian placenta has evolved mechanisms that help buffer the fetus from short-term fluctuations in maternal diet and energy status [29]. In order to avoid this buffering mechanism, most of the studied protocols reduce maternal nutritional intake to 50–60% of the normal amount, exerting a significant impact on fetal growth and development through high levels of food restriction [7]. Moderate or low levels of food restriction may better mimic the clinical features of malnourished women, but few studies have investigated the effects of moderate food restriction during pregnancy on embryonic development.

In addition to maternal nutritional interventions, the smaller size of the embryonic mammary gland presents challenges for the study of mammary gland development. The most visual method of viewing mammary gland development is the whole mount, a method of viewing a three-dimensional overview of the mammary gland, which provides a dense ductal epithelial structure within the complete mammary gland [30]. The whole mount requires the complete mammary gland to be isolated from the skin of the mouse and spread out as naturally as possible, which is more challenging for embryonic and newborn mice. In earlier studies, the results of the whole-mount analysis were difficult to quantify and were only used as a display image in the studies [31]. The complex ducts of the mammary gland in puberty can be evaluated in terms of the area covered and the denseness of the ducts observed visually. However, this unquantifiable observation is difficult to evaluate in the primary mammary gland, which has only 10–15 branches at birth. Jason P. Stanko et al. reported the use of Sholl analysis, an ImageJ plug-in for neuronal analysis, to quantify whole-mount results of the mammary gland [32]. The Sholl analysis creates a series of concentric rings based on a custom center (origin of the mammary duct) and extends to the most distal portion of the branch (enclosing radius). The Sholl analysis plug-in calculates the number of intersections that occur on each ring and then returns a Sholl regression coefficient (k), which is a measure of the rate of decay of the epithelial branches. In Sholl analysis, the sum inters (N) is the number of intersections of multiple concentric circles centered on the primary ducts with the ducts, reflecting the complexity of the mammary gland. The sholl regression coefficient (k) is a measure of the distal mammary branch complexity, which is close to 0, indicating more complex and well-developed distal mammary branches. Branch density is calculated using the formula N/MEA. Sholl analysis provides a valid quantitative measure of mammary branch complexity and has become a reliable method for studying mammary gland development. Mammary gland development in embryonic mice can be evaluated through a combination of fine dissection and whole-mount and Sholl analysis.

Different levels of maternal nutritional restriction may have different effects on embryonic mammary gland development due to different maternal nutritional buffering and stem cell responses to nutrition. To investigate this, we established a pattern of nutritional restriction on mammary gland development during embryonic period by setting 100%, 90%, 80%, 70% and 60% diet intakes for female mice during pregnancy. The objective of our study was to investigate the effect of maternal gradient nutritional restriction on mammary gland development in offspring and provide a reference for the amount of maternal nutritional restriction. 

## 2. Materials and Methods

### 2.1. Animals and Experimental Design

Sixty female 8-week-old CD-1(ICR) mice were provided by Vital River Laboratory Animal Technology Co., (Beijing, China) and mated with males of similar age. Each male mouse was put in a cage with 1 female mouse. Mating of mice was demonstrated by the presence of vaginal plugs. Female mice were individually housed after the discovery of the vaginal plugs and recorded as day 0 of gestation. All mice in our study were fed commercially available irradiated sterile growth and reproduction diets for experimental mice (SFS9112, Xietong Biotechnology, Yangzhou, China). To reduce the impact of nutritional restriction on early embryonic growth, it began on the ninth day of pregnancy. Pregnant mice were divided into five groups (*n* = 12): the 100% group was fed ad libitum (control group), and the 90%, 80%, 70% and 60% groups were fed 90%, 80%, 70% and 60% of the ad libitum food weight daily, respectively. The ad libitum group was mated one day earlier and their intake was used as the basis multiplied by 90%, 80%, 70% and 60% as the feed intake for the gradient nutrient restriction. The weight of the mice was recorded daily. The number of litters as well as the weight of the female mice and offspring were recorded on the day of delivery.

### 2.2. Body Fat Percentage Assay

On the day of delivery, whole body image and body fat percentage were evaluated in vivo using dual-energy X-ray absorptiometry (DEXA) on an InAlyzer (Medikors Co., Seongnam, Republic of Korea). Female mice and female offspring were anesthetized using isoflurane (RWD, Shenzhen, China) and placed on a scanner bed and operated according to the instructions. After in vivo imaging, female offspring mice were euthanized using CO_2_.

### 2.3. Collection and Preservation of Mammary Glands

The mammary glands were removed immediately after euthanasia, the #4 inguinal mammary glands were placed on slides and immersed in Carnoy’s solution for whole mount, and the other mammary glands were stored at −80 °C for real-time quantitative polymerase chain reaction (qPCR).

### 2.4. Mammary Whole Mount

The mammary glands were fixed in Carnoy’s solution (60% absolute ethanol, 30% chloroform, 10% glacial acetic acid) for 4 h and then placed sequentially in ethanol at 100%, 70%, 50% and 10% concentrations for 15 min each. After soaking in deionized water for 5 min, the mammary glands were stained using carmine alum solution (1 g carmine alum, 2.5 g aluminum potassium sulfate in 500 mL dH_2_O) for 4 h. The stained mammary glands were soaked for 5 min using distilled water, then sequentially soaked in 70%, 95% and 100% alcohol, each concentration for 15 min. The mammary glands were placed in xylene for 12 h for transparency and then sealed with neutral resin. Whole-mount slices of mammary glands were sectioned for image acquisition using an upright microscope (Nikion, Japan).

### 2.5. RNA Extraction and qPCR

RNA from offspring mammary glands was extracted using RNA-easy Isolation Reagent (R701-01, Vazyme Biotech, Nanjing, China) according to the instructions. RNA quality was evaluated by 1% agarose gel electrophoresis, while the purity of the total RNA was determined by NanoDrop 2000 (NanoDrop, ThermoFisher Science, Waltham, MA, USA). The genomic DNA was removed from each RNA sample and reverse-transcribed into cDNA using an Evo M-MLV Mix Kit (Accurate Biology, AG11728, Hunan, China). Then qPCR was performed using a SYBR Green Premix Pro Taq HS qPCR Kit (Accurate Biology, AG11701, Hunan, China) with a LightCycler 96 Instrument (Roche, Basel, Switzerland). The reaction program was set to pre-denaturation at 95 °C for 30 s, followed by denaturation at 95 °C for 5 s and extension at 60 °C for 30 s, for a total of 40 cycles, with each reaction repeated 3 times. The primer sequences are shown in Table 1. The amplification efficiency and the specificity of the amplified products of each primer pair were verified using standard curves and melting curves, respectively. The mRNA expression of each sample was normalized relative to the expression of glyceraldehyde 3-phosphate dehydrogenase (GAPDH). Relative gene expression levels of each target gene were analyzed using the 2^−ΔΔct^ method.

### 2.6. Statistical Analysis

We performed Sholl analysis on mammary whole-mount results according to the method described in a previous study [32]. Mammary gland whole-mount analysis was performed using ImageJ 2.1 software, and the Sholl analysis plugin 4.0.1 for ImageJ was used for Sholl analysis. The distance from the primary ducts to the most distal end of the mammary epithelium (enclosing radius) and the mammary epithelial area (MEA) were measured using ImageJ. The Sholl analysis was performed with the primary duct as the center, the enclosing radius as the ending radius and a radius step size of 0.02 mm. Since the mammary ducts in newborn mice are less developed and farther away from the mammary lymph nodes, the area occupied by the mammary lymph nodes was not calculated in Branch density.

Body weight, body fat, litter size, Sholl analysis results and gene expression were analyzed using one-way ANOVA in the ad libitum feeding, 90%, 80%, 70% and 60% groups. One-way ANOVA was performed using IBM spss 25 (Armork, NY, USA), with Sidak correction for multiple testing. Body weight, body fat, litter size and gene expression data were presented as the mean ± the standard deviation (SD). Principal component analysis (PCA) was performed on enclosing radius, MEA, sum inters and k from the results of Sholl analysis in all groups. PCA was performed using the FactoMineR and factoextra packages in R4.2.1, and PCA biplot figures were generated. The enclosing radius of each group in the Sholl analysis results were regressed against MEA, sum inters and k. Linear regression analysis of the mammary Sholl analysis was performed using simple linear regression in GraphPad Prism software 9.1.0 (San Diego, CA, USA).

## 3. Results

**Figure 1:**
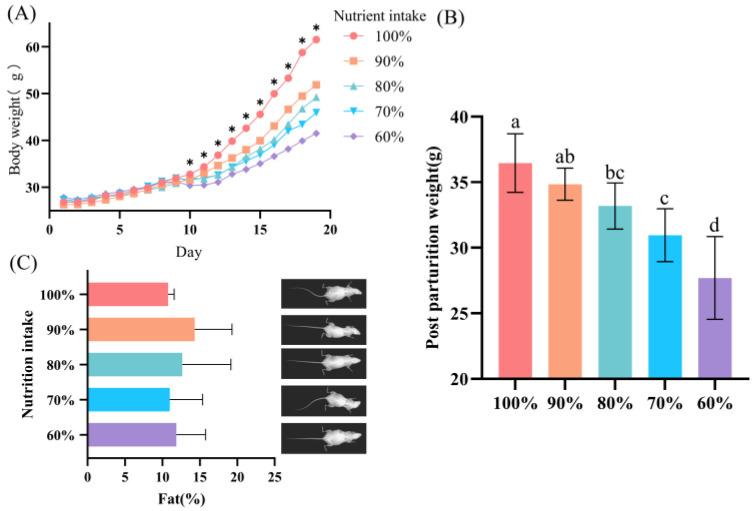
Body weight and body fat of gestational nutritional restriction female mice. (**A**) Body weight of female mice during gestation. (**B**) Postpartum weight of female mice. (**C**) Body fat percentage and X-ray images of female mice after parturition. Identical letters are not significant difference (*p* > 0.05), while different letters indicate significant difference (*p* < 0.05), determined by one-way ANOVA followed by a Sidak multiple-comparison test. Bar charts represent mean, error bars represent SD. Different colors represent different groups. Asterisk stands for *p* < 0.05 and no asterisk means no significant difference.

After nutrient restriction management, a significant difference in body weight was observed in mice from day ten of pregnancy, and the difference persisted until the end of gestation (*p* < 0.05; Figure 1A). After parturition, the adult female mice showed a significant decrease in body weight compared to the control group (*p* < 0.05), except for the 90% group (*p* > 0.05; Figure 1B). However, there was no significant difference in body fat percentage in adult female mice after parturition (*p* > 0.05; Figure 1C).

**Figure 2:**
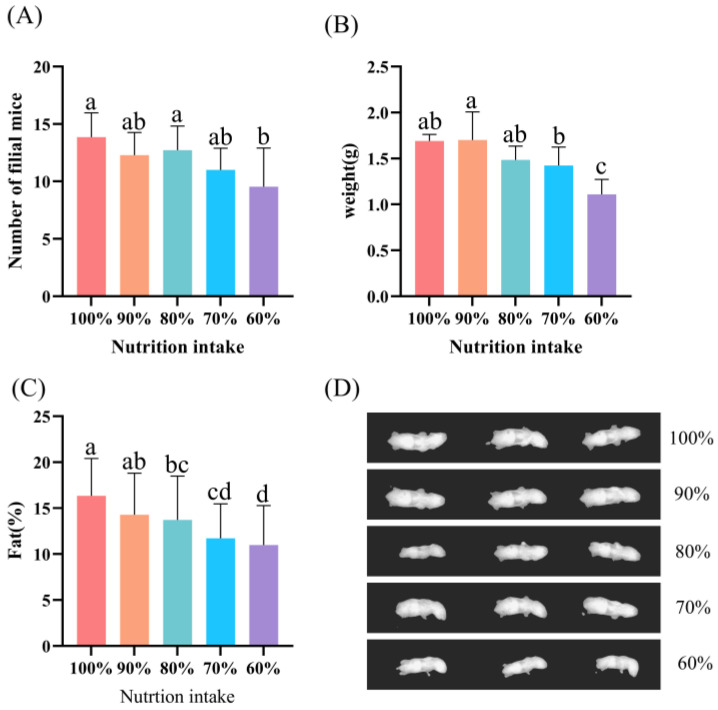
The number, body weight, body fat and X-ray images of maternally nutritionally restricted offspring. (**A**) Litter size in different nutrient-restricted groups. (**B**) Birth weight of offspring mice in different nutritionally restricted groups. (**C**) Body fat percentage of offspring mice in different nutrient restriction groups. (**D**) X-ray images of offspring mice in different nutritionally restricted groups. Identical letters are not significant difference (*p* > 0.05), while different letters indicate significant difference (*p* < 0.05), determined by one-way ANOVA followed by a Sidak multiple-comparison test. Bar charts represent mean, error bars represent SD. Different colors represent different groups.

When the nutritional intake was only 60% of the normal intake, a significant decrease in litter size was observed compared to the control group (*p* < 0.05; Figure 2A), while the individual offspring weight was significantly lower than that of the other groups (*p* < 0.05; Figure 2B). The body fat percentage of the offspring was significantly higher in the control group than in the 80%, 70% and 60% groups (*p* < 0.05; Figure 2C,D).

**Figure 3:**
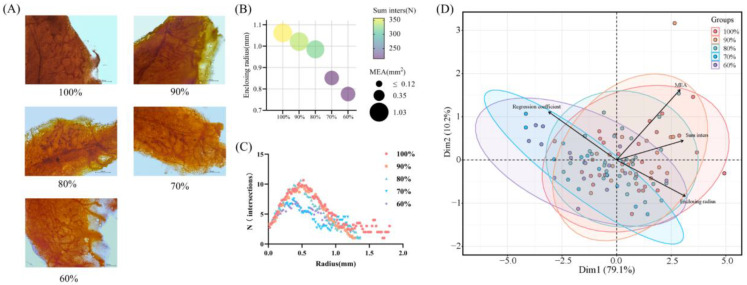
Whole-mount and Sholl analysis of #4 inguinal mammary glands from maternally restricted offspring. (**A**) Whole-mount image of mammary glands in different nutrient-restricted groups. (**B**) Bubble plot of mammary gland Sholl analysis. (**C**) Linear Sholl plots of mammary glands. (**D**) Principal component analysis (PCA) biplot of mammary gland Sholl results.

The mammary whole-mount images are shown in Figure 3A. For the enclosing radius, a significant increase was observed in the control group compared to the 70% and 60% groups (*p* < 0.05), while a significant increase was observed in the 90% group compared to the 60% group (*p* < 0.05; Table 2). MEA did not differ in the control, 90% and 80% groups (*p* < 0.05), while it was significantly lower in the 70% and 60% groups than in the former three groups (*p* < 0.05; Table 2). Sum inters were significantly higher in the control, 90% and 80% groups than in the other two groups (*p* < 0.05; Table 2). The k of 70% and 60% were significantly higher than the other three groups (*p* < 0.05; Table 2). Branching density was not significantly different among the groups (*p* > 0.05; Table 2). Consistent with the results in Table 2, an identifiable change in mammary gland development was observed from the 80% group to the 70% group in Figure 3B. Figure 3C shows the number of intersections of each concentric circle with the mammary ducts in the Sholl analysis. The control group had the longest duct extension distance. At a radius of 0.5 mm, the control, 90% and 80% groups reached the highest number of intersections with a similar peak, all higher than the 70% and 60% groups.

In order to further investigate the reasons for the dramatic decline in offspring mammary development from the 80% group to the 70% group, we performed a PCA (Figure 3D) of the mammary whole-mount results (enclosing radius, MEA, sum inters and k). After dimensionality reduction, the data points in the control, 90% and 80% groups were nearer to each other, forming visible distance differences with the 70% and 60% groups, indicating that a massive reduction in mammary gland development in the offspring occurs when the maternal nutritional limit is reduced from 80% to 70%. The PCA bipartite plot shows the scores and loadings of the first two components (dim1 and dim2), revealing the projection of the observed indicators on a space with dim1 and dim2 as axes. In our study, the indicators of mammary gland development were explained by 79.1% of dim1 and 10.2% of dim2, respectively. The variable with the highest weight in the first principal component is the enclosing radius, indicating that the main reason for the difference in distance from the 80% to the 70% group in the mammary glands was the change in enclosing radius. A positive correlation between enclosing radius and MEA and sum inters and a negative correlation with k are presented in the PCA biplot.

**Figure 4:**
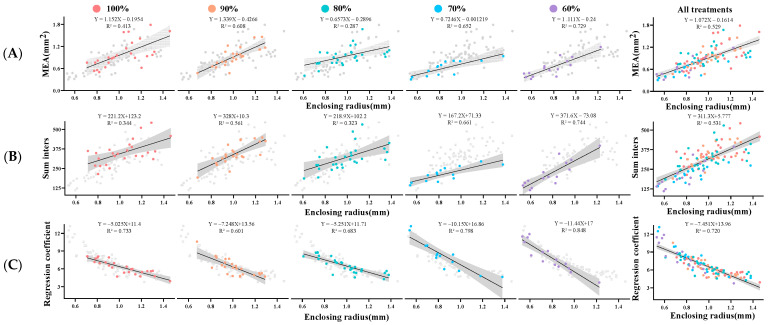
Regression analysis of mammary whole-mount results from maternally restricted offspring. Regression analysis between (**A**) mammary enclosing radius and mammary epithelial area (MEA), (**B**) mammary enclosing radius and sum inters and (**C**) mammary enclosing radius and Sholl regression coefficient (k). The dots represent individual measured values, the black lines represent linear regressions, and the gray areas represent their 95% confidence intervals.

To analyze the effect of the enclosing radius, which has the highest weight in PCA, on the pattern of mammary gland development in the offspring, we performed a regression analysis of the whole-mount results (Figure 4). In the regression analysis of the enclosing radius with MEA, the 90% and 60% groups had larger slopes compared to the control group, while the 80% and 70% groups had smaller slopes. In the regression analysis of the enclosing radius versus sum inters, as maternal nutritional restriction increased, the slope first increased in the 90% group, then gradually decreased in the 80% and 70% groups and then showed an increase in the 60% group. In the regression analysis of the enclosing radius versus k, the slope of each group is less than the control group, with the 60% group having the lowest slope.

**Figure 5:**
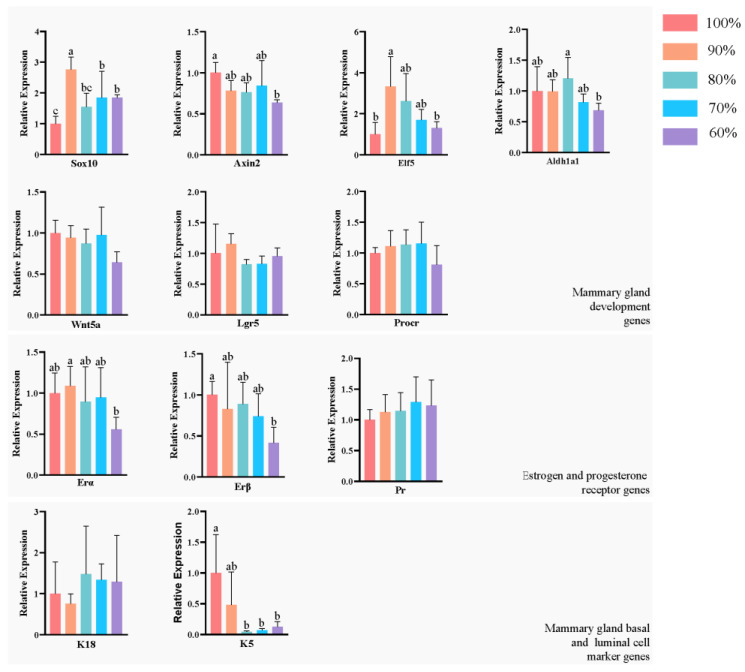
Mammary gland proliferation- and hormone-related gene expression in offspring. Identical letters or no letters are not significant difference (*p* > 0.05), while different letters indicate significant difference (*p* < 0.05) determined by one-way ANOVA followed by a Sidak multiple-comparison test. Bar charts represent mean, error bars represent SD. Sox10: SRY (sex-determining region Y)-box 10. Axin2: Axis inhibition protein 2. Elf5: E74-like factor 5. Aldh1a1: aldehyde dehydrogenase family 1. Wnt5a: wingless-type MMTV integration site family, member 5A. Lgr5: leucine-rich repeat-containing G-protein-coupled receptor 5. Procr: protein C receptor. ERα: estrogen receptor 1 (alpha). ERβ: estrogen receptor 2 (beta). Pr: progesterone receptor. K18: keratin 18. K5: keratin 5.

We analyzed the expression of development-related genes (Sox10, Axin2, Elf5, Lgr5, Wnt5a, Aldh1a1, Procr), mammary basal cell marker genes (K5), mammary luminal cell marker genes (K18), estrogen (ERα,ERβ) and progesterone receptor (PR) genes in the mammary glands (Figure 5) by one-way ANOVA followed by a Sidak multiple-comparison test. In the 90% group, Sox10 expression was significantly higher than in the other four groups (*p* < 0.05), and Elf5 was significantly higher than in the control and 60% groups (*p* < 0.05). Sox10 was significantly lower in the control group than in the 90%, 70% and 60% groups (*p* < 0.05), and Axin2 was significantly higher in the control group than in the 60% group (*p* < 0.05). Aldh1a1 was significantly higher in the 80% group than in the 60% group (*p* < 0.05). The expression of K5 was significantly higher in the control group than in the 80%, 70% and 60% groups (*p* < 0.05). In the 60% group, ER1 was significantly lower than in the 90% group (*p* < 0.05) and ER2 was significantly lower than in the control group (*p* < 0.05). The expression of other genes did not differ significantly among the groups (*p* > 0.05).

## 4. Discussion

Nutritional deficiencies during gestation cause irreversible effects in fetal organs [33], but nutritional deficiency research on embryonic mammary gland development remains vacant. The impairment of mammary gland development at this phase may directly lead to delayed fetal mammary gland development in adulthood [9]. The small size of the mammary gland, which is difficult to observe, and the buffering through the placenta, which reduces the impact of nutritional fluctuations in the embryo, present challenges for the study of mammary gland development during this period. We performed a quantitative study of the mammary glands using whole mount combined with Sholl analysis and further analyzed the developmental pattern of the mammary gland by PCA and regression analysis. Through maternal gradient nutrient limitation, we established a pattern of offspring mammary gland development and revealed stem cell-related gene expression through a gradient reduction in maternal nutrient intake from 100% to 60% during gestation. The main findings of the study were: (1) Mild maternal nutritional restriction (90–70% of ad libitum intake) did not affect offspring weight, while body fat percentage was more sensitive to nutritional restriction (lower at 80% ad libitum feeding). (2) A precipitous drop in mammary development and altered developmental patterns occurred when nutritional restriction ranged from 80% to 70% of ad libitum intake. (3) Mild maternal nutritional restriction (90% of ad libitum intake) promoted mammary development-related gene expression.

Inadequate nutrition during pregnancy can have an impact on maternal and fetal health, most notably in the form of weight loss [34]. In our study, differences in body weight of female mice emerged from the tenth day of gestation after nutritional restriction. After delivery, maternal mice in the 80% group lost significant body weight, while body fat percentage was not affected. For offspring, body fat percentage decreased first when nutritional intake was 80% of ad libitum, and weight loss occurred when it was 60%. Our result is similar to a previous study, which found no significant change in offspring birth weight during gestation for a maternal restriction to 75% ad libitum feeding [28]. A reduction in offspring body weight occurs when nutritional restriction reaches 60% or less of the ad libitum intake [34,35]. It seems that embryonic body fat percentage is more susceptible than body weight when faced with nutritional constraints. Mammary ducts and epithelium need to be embedded in the mammary stroma for growth, which is composed of homogeneous adipose tissue [36]. In studies on obesity, there is a strong association between mammary fat pads and obesity [37]. Although mammary fat pad and body fat have not been studied in studies on nutritional restriction, the possibility exists that a decrease in whole body fat percentage may affect mammary fat pad development.

To assess mammary gland development, we performed a Sholl analysis on the mammary glands of offspring with different levels of nutritional restriction. Based on the Sholl analysis reported in the previous study [32], we innovatively performed PCA analysis and regression analysis on the results of the Sholl analysis to explore the developmental pattern of mammary glands. We found a dramatic decrease in mammary gland development when nutritional restriction was dropped from 80% to 70% of ad libitum intake. In contrast, there was no significant difference in the effect of normal feeding versus 90% and 80% of ad libitum feeding on mammary gland development. We hypothesize that the dramatic delay in mammary gland development may be due to the buffering of embryonic nutrients by the placenta as a “nutrient sensor” [29]. For maternal nutritional restriction to 90% and 80% of normal intake, the buffering mechanism in the maternal body mitigates the effect of nutrition on fetal mammary development, and as the intake decreases to 70%, the maternal buffering limit is exceeded, resulting in delayed mammary development. Inconsistently with our results, the nutritional intake of sows being restricted to 70% of ad libitum intake had no effect on the weight of mammary parenchyma, fat content, protein content and DNA content of the offspring [38]. The weight, DNA content and other methods used in their study to evaluate the mammary glands do not provide a complete view of the development of the mammary ducts compared to the whole mount. In addition, the species may also be responsible for the discrepancy between their results and our findings.

From the results of the PCA analysis, we determined that the variable with the greatest weight is the enclosing radius. This suggests that the dramatic decrease in mammary development from the 80% to the 70% group was mainly caused by changes in the enclosing radius. To analyze the developmental pattern of the mammary glands, we performed a regression analysis of the enclosing radius with MEA, sum inters and k. Our study found a positive regression relationship between the enclosing radius and MEA and sum inters and an inverse regression relationship with k. This suggests that as the distance of the terminal duct from the primary duct increases, the mammary gland will cover a larger area with more complex branching, while the terminal decay will be slower. Similar to our results, the similar trend of the mammary longitudinal extension distance with the mammary epithelial area was found in a previous study [39].

We found that compared to controls, mild nutritional restriction (90% of ad libitum intake) had larger regression coefficients in regression analyses with MEA and sum inters and smaller regression coefficients with k. This suggests that offspring with mild maternal nutritional restriction have better potential for mammary gland development. When nutrition was restricted to 80% of ad libitum feeding, the regression coefficients of the enclosing radius and MEA reflected reduced mammary epithelial area growth potential, despite no difference in mammary gland developmental indicators compared to the control group. Interestingly, the regression coefficients of MEA, sum inters and k all showed greater absolute values when the nutritional restriction was 60% of the ad libitum intake. At this level of nutritional restriction, the enclosing radius already showed a significant shortening and, therefore, mammary gland development slowed down.

Sox10, Axin2 and Elf5 have been shown to function as key genes in embryonic mammary gland development. Sox10 is expressed in fetal mammary gland stem cells during embryonic mammary gland development and plays a central role in mammary gland development [40,41]. Axin2, a target gene of the Wnt/β-catenin pathway, has been used as a marker of functional stem cells in the mammary gland in a lineage-tracing approach [18]. Elf5 is required for the proliferation and differentiation of mammary epithelial cells in embryonic mouse mammary glands [42]. We found that mild nutritional restriction (90% of ad libitum intake) increased the gene expression of Sox10 and Elf5, suggesting a positive effect on mammary gland development. In a previous study on dietary control, alternate-day nutritional restriction was proven to increase the activity of tissue-specific stem cells and had positive implications for life extension [43]. Combined with our regression analysis of whole-mount results, our results suggest that mild maternal nutritional restriction does not impair offspring mammary development and may even increase offspring mammary growth potential by increasing the expression of stem cell-related genes. In addition, 60% of ad libitum feeding reduced Axin2 expression, suggesting that high levels of nutritional restriction inhibit mammary stem cell development and mammary gland development. Consistent with our results, in a study of high levels of maternal gestational nutritional restriction (50% of ad libitum feeding), the ability to differentiate neural progenitor cells was decreased [44]. These results suggest that stem cell activity in the embryonic mammary gland is related to the level of maternal nutritional restriction, with mild nutritional restriction contributing to stem cell-associated gene expression and high nutritional restriction inhibiting them. K5 is a known marker gene in the myoepithelial/basal layer of the mammary gland [45]. Our study shows that a decrease in K5 gene expression occurs in the basal lamina of the mammary glands when nutrition is restricted to 80% of ad libitum feeding. Combined with regression analysis, our results showed that the expansion potential of mammary basal and mammary gland area was affected by maternal nutritional restriction up to 80% of ad libitum feeding, despite no significant difference in the results of whole-mount analysis.

Embryonic mammary gland development is considered to be hormone-nondependent, and previous studies have demonstrated that embryonic mammary glands are able to develop in mice lacking estrogen (ER-α and ER-β) and progesterone receptors [9,46,47]. After birth, especially during puberty, the mammary glands are stimulated by these hormones to develop rapidly. Estrogen is required for the branching of the mammary ducts during puberty, and estrogen and progesterone are required for lobuloalveolar development during pregnancy. In our study, ER-β receptor expression appeared to be reduced when nutritional restriction reached 60% of the ad libitum intake. This suggests that high levels of maternal nutritional restriction may affect the development of offspring mammary estrogen receptors whose impairment may have further effects on mammary development during puberty.

## 5. Conclusions

In conclusion, our results suggest that mild maternal nutritional restriction (90% of ad libitum intake) during gestation contributes to increased embryonic mammary gland development. When nutritional restriction ranges from 80% to 70% of ad libitum intake, mammary gland development decreases dramatically, and changes in developmental patterns occur.

## Figures and Tables

**Table 1:** Nucleotide sequences of real-time PCR primers.

Gene ^1^	Accession Number	Primer Sequence, 5′→3′
Sox10	NM_011437.1	F: CTGAGCTCAGCAAGACACTAG
		R: GTTGGTACTTGTAGTCCGGATG
Axin2	NM_015732.4	F: AGCCTAAAGGTCTTATGTGGCTA
		R: ACCTACGTGATAAGGATTGACT
Elf5	NM_001145813.1	F: GAGCATCAGACAGCCTGTGA
		R: CCATTCCAGGATGCCACAGT
Aldh1a1	NM_001361503.1	F: CAGTGAGCGGCAAGAAA
		R: GGAGAGCCAATCTGGAAAG
Wnt5a	NM_001256224.2	F: GAATCCCATTTGCAACCCCTCACC
		R: GCTCCTCGTGTACATTTTCTGCCC
Lgr5	NM_010195.2	F: GCTCAACTCTCTCTGTTTCCTCA
		R: GGTGAGGTTTAGCAAAGAGGAGA
Procr	NM_011171.2	F: CTCTCTGGGAAAACTCCTGACA
		R: CAGGGAGCAGCTAACAGTGA
ERα	NM_001302531.1	F: TGTGTCCAGCTACAAACCAATG
		R: CATCATGCCCACTTCGTAACA
ERβ	NM_010157.3	F: TGTGTGTGAAGGCCATGATT
		R: TCTTCGAAATCACCCAGACC
Pr	NM_008829.2	F: GGTCCCCCTTGCTTGCA
		R: CAGGACCGAGGAAAAAGCAG
K18	NM_010664.2	F: CTGGGGTGGCTCTGTGGGGT
		R: GGCTTCCAGACCTTGGACTTCCTCT
K5	NM_027011.3	F: GTACCAGACCAAGTATGAGGAGC
		R: CCTCTGGATCATTCGGTTCATCT
Gapdh	NM_001289726.2	F: TGTGTCCGTCGTGGATCTGA
		R: TTGCTGTTGAAGTCGCAGGAG

^1^ Sox10: SRY (sex-determining region Y)-box 10. Axin2: Axis inhibition protein 2. Elf5: E74-like factor 5. Aldh1a1: aldehyde dehydrogenase family 1. Wnt5a: wingless-type MMTV integration site family, member 5A. Lgr5: leucine-rich repeat-containing G-protein-coupled receptor 5. Procr: protein C receptor. ERα: estrogen receptor 1 (alpha). ERβ: estrogen receptor 2 (beta). Pr: progesterone receptor. K18: keratin 18. K5: keratin 5. Gapdh: glyceraldehyde-3-phosphate dehydrogenase.

**Table 2:** Sholl analysis of mammary grands from maternally restricted offspring.

	100%	90%	80%	70%	60%	SEM	*p*-Value
Enclosing radius (mm)	1.062 ^a^	1.021 ^ab^	0.985 ^ab^	0.851 ^bc^	0.776 ^c^	0.022	<0.01
MEA (mm^2^) ^1^	1.033 ^a^	1.010 ^a^	0.937 ^a^	0.615 ^b^	0.622 ^b^	0.036	<0.01
Sum inters (n)	357.07 ^a^	337.44 ^a^	317.97 ^a^	213.57 ^b^	215.33 ^b^	8.80	<0.01
k ^2^	6.071 ^a^	6.199 ^a^	6.539 ^a^	8.232 ^b^	8.122 ^b^	0.179	<0.01
Branching density (N/mm^2^)	369.417	367.253	343.607	364.029	349.753	6.147	0.425

The different letters indicate statistically significant differences (*p* < 0.05). ^1^ MEA: mammary epithelial area. ^2^ k: Sholl regression coefficient.

## Data Availability

If requested, the authors guarantee that the data supporting the results reported in this article may be provided.
